# Interventional study to improve adherence to phosphate binder treatment in dialysis patients

**DOI:** 10.1186/s12882-019-1334-x

**Published:** 2019-05-17

**Authors:** Bodil Jahren Hjemås, Katrine Bøvre, Liv Mathiesen, Jonas Christoffer Lindstrøm, Kathrin Bjerknes

**Affiliations:** 1 Hospital Pharmacies Enterprise, South Eastern Norway, Stenersgate 1, PB. 79, 0050 Oslo, Norway; 2 Department of Pharmaceutical Biosciences, School of Pharmacy, University of Oslo, Oslo, Norway; 3 Health Services Research Units, Akershus University Hospital, Institute of Clinical Medicine, University of Oslo, Oslo, Norway

**Keywords:** Adherence, Beliefs about medicines, Hemodialysis, Hyperphosphatemia, Phosphate binder, Pharmacist led education

## Abstract

**Background:**

Adherence to phosphate binder treatment is important to prevent high serum phosphate level in chronic dialysis patients. We therefore wanted to investigate patient knowledge, beliefs about and adherence to phosphate binders among these patients and assess whether one-to-one pharmacist-led education and counselling enhance adherence and lead to changes in serum phosphate levels.

**Methods:**

A descriptive, interventional, single arm, pre-post study was performed at a hospital in Norway, including chronic dialysis patients aged 18 years or more using phosphate binders. The primary end-point was change in the proportion of patients with serum phosphate below 1.80 mmol/L and the secondary end-points included change in the patient’s knowledge, beliefs and adherence after the intervention measured by completion of questionnaires ‘Patient Knowledge’, Medication Adherence Report Scale (MARS− 5) and Beliefs about Medicines Questionnaire (BMQ). Data was collected both prior to and after one-to-one pharmacist-led education and counselling about their phosphate binders. Other medicines used by the patient was also registered.

**Results:**

A total of 69 patients were enrolled in the study. After intervention, the probability of serum phosphate being below the target threshold 1.80 mmol/L (5.58 mg/dL) increased, although no significant change in mean serum phosphate levels was seen. On the other hand, the knowledge regarding phosphate binder treatment and the patients’ beliefs about the necessity of the treatment increased, while the concerns decreased (BMQ). This effect did not lead to increase in self-reported adherence measured by MARS-5. However the scores were high before the intervention.

**Conclusions:**

Short term one-to-one individualized pharmacist-led education and counselling about phosphate binders increased the probability of serum phosphate concentrations being below the target threshold level 1.80 mmol/L (5.58 mg/dL), although not statistically significant. However, it did not decrease the mean serum phosphate level or increase the patients’ self-reported adherence. The patients increased their knowledge about the phosphate binder and their understanding of adherence, and were less concerned about the side effects of the medication.

**Trial registration:**

ISRCTN52852596, registered 11 April 2019. The trial was registered retrospectively.

## Background

Hyperphosphatemia is present in the majority of dialysis patients and is associated with increased risk of cardiovascular mortality [1–3]. Drug treatment with phosphate binders is therefore indicated in most chronic dialysis patients, but adherence is often poor [4, 5].

In a systematic review including 44 publications, the prevalence of medication non-adherence varied from 12.5–98.6% [6]. Different definitions and methods as well as many factors influencing adherence made the picture complex. Factors associated with non-adherence were both patient related, medication related and disease related, including illness interfering family life, longevity of hemodialysis, depressive symptoms and complexity of medication regimen.

Adherence to medication regimes in patients with chronic kidney disease (CKD) has been found to correlate with beliefs about medicines in a study using the validated tool Beliefs about Medicines Questionnaire (BMQ) [7]. The Medication Adherence Report Scale (MARS) has been developed to explore self-reported adherence [8]. These tools were applied in a previous study including 160 patients with CKD. Adherence was associated positively with family and work status as well as patient’s concerns about medicine measured by BMQ [9].

The correlation between medication beliefs measured by BMQ, self reported adherence measured by MARS and serum phosphate in hyperphosphatemic haemodialysis patients was studied in a randomized controlled trial [10]. They found that medication necessity beliefs explained the variance of serum phosphate and self-reported adherence. The authors pointed out that “Dialysis patient’s medication beliefs are potentially modifiable targets for future interventions”.

Studies including different interventions like one-to one educational sessions, telephone-calls, demonstration and leaflets has to variable degrees been successful in improving adherence to phosphate binders and lowering of serum phosphate levels [4, 11–14]. The lack of consistence in results between the studies as well as the overall complex picture of numerous factors influencing adherence, underlines the need of further research to increase the evidence for practice in this area. Pharmacist-led education and counselling can be one strategy to modify beliefs about medications and hence increase adherence to phosphate binder treatment in dialysis patients.

The aim of this study was to investigate knowledge, beliefs about and adherence to phosphate binders among chronic dialysis patients and to assess whether one-to-one pharmacist-led education and counselling could improve phosphate binder adherence and lead to changes in serum phosphate levels.

## Methods

A descriptive, interventional, single arm, pre-post study was performed at Akershus University Hospital in Norway, recruiting patients at the dialysis centre during May and June 2017. Chronic dialysis patients using phosphate binders received written and oral information about the study. Eligibility criteria were: 1) age > 18 years, 2) receiving chronic dialysis two to four times a week for at least 5 months, 3) life expectancy > 5 months, 4) using at least one self-administered phosphate binder, 5) able to speak, read and write Norwegian, and 6) able to give informed consent. Demographic characteristics, medical and pharmacological data were collected from the medical record and by face-to-face interview.

The collection of data was done twice; before and after the intervention. The primary end-point was change in the proportion of patients with serum phosphate below 1.80 mmol/L (5.58 mg/dL). The secondary end-points were to assess whether the patients’ knowledge, beliefs and adherence changed. In addition, other medicines used by the patient were registered to see whether the use of psycholeptic and/or psychoanaleptic medicine correlated with serum phosphate levels, adherence and beliefs.

Pre-dialytic (not fasting) values of serum phosphate is monthly monitored at the hospital and registered in the medical record. In the study, data on serum phosphate levels for 5 months prior to the intervention, and 5 months after were drawn from the medical records for included patients.

A serum phosphate target threshold < 1.80 mmol/L (5.58 mg/dL) was set in consistence with the hospital guidelines referring to UpToDate and the Kidney Disease Outcomes Quality Initiative guidelines (KDOQI) [15, 16].

One study pharmacist executed a half-hour one-to-one intervention, comprising a single session education and personalized counselling about the use of phosphate binders, as outlined in Table 1. The intervention was performed at the hospital dialysis centre during dialysis treatment, 2–3 weeks after inclusion of each patient. A semi-structured counselling guide, partly based on the script used in the study by Van Camp et al [11], was used in the education and counselling. In addition, an educational leaflet based on this guide was offered to the patients. This included information about phosphate and functions in the body, foods containing phosphate, how we can get too much phosphate and what consequences this might have. It also contained information about the phosphate binder: effect, importance, practical information regarding intake, possible side effects and interactions with other medicines.

**Table 1:**
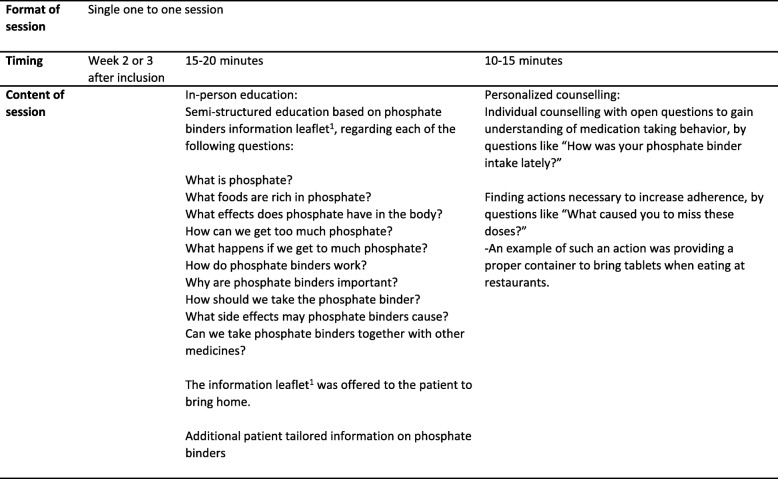
Key elements of the pharmacist-led education and counselling. ^1^Educational leaflet partially based on the phosphate binders pamphlet used by van Camp et al. [11]

During the study period, the usual care regarding education and counselling specific to phosphate and phosphate binder treatment was continued in the dialysis unit. This included physician counselling and information (oral and written) related to serum phosphate monitoring and when starting or changing phosphate binder treatment.

The participants were asked to answer the three questionnaires (‘Patient Knowledge’, MARS-5 and BMQ) twice; at the time of inclusion and 3–4 weeks after the intervention. The patients answered the questionnaires during dialysis treatment.

**Fig. 1:**
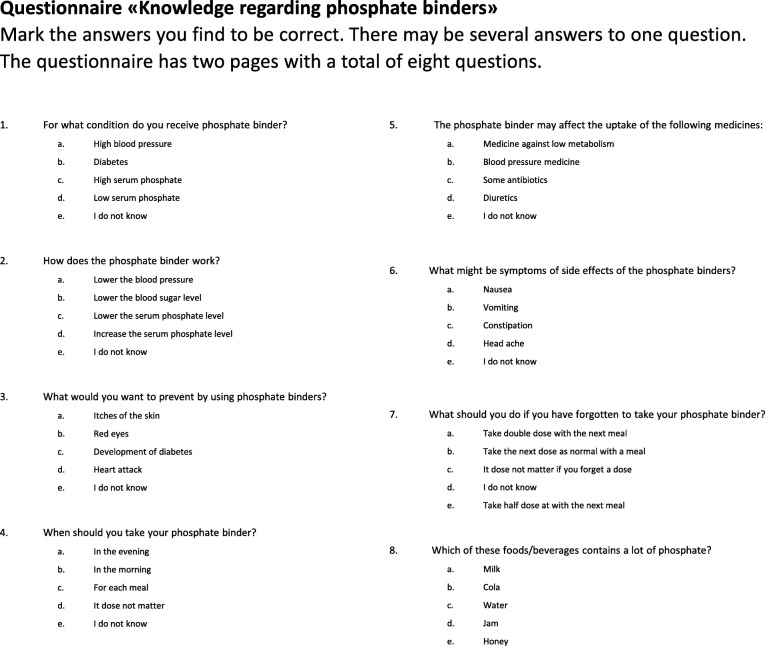
The multiple choice ‘Patient Knowledge’ questionnaire. One point was given for each correct answer giving a potential total score of 16.0

The competence in the study group regarding phosphate binder (‘Patient Knowledge’) was assessed by an eight-item multiple-choice questionnaire based on the questionnaire developed by Eide et al [17], as outlined in (Fig. 1). The questionnaire was evaluated by the hospital user representative and one dialysis patient. The usability was assessed by letting two other dialysis patients fill out the questionnaire and state if they understood the questions or not. Some minor changes in format was included before start of the main study.

Adherence was measured with the MARS-5 [18] and permission to use the scale was provided by Professor Robert Horne. The original scale consists of general statements about medicines, and in this study the statements were specified with the term phosphate binder. The MARS-5 is a 5-item self-report scale and the responses are scored on a 5-point Likert- scale where 1 = always, 2 = often, 3 = sometimes, 4 = rarely, and 5 = never. Total scores for the whole questionnaire range from 5 to 25, with higher scores indicating higher self-reported adherence.

The patients’ perception of medication was measured by BMQ, consisting of two sections, BMQ-Specific, which assesses beliefs about the necessity and concerns for personal medicines, and BMQ-General, which assesses general beliefs about overuse and harm of medicines. Responses are scored on a 5-point Likert-scale. Scores for the individual items within each scale were summed to give a total score, ranging from 6 to 30 for the ‘Necessity scale’, 5 to 25 for the ‘Concerns scale’ and 4 to 20 for the ‘Overuse and Harm scales’. Higher scores indicate stronger beliefs in the concepts represented by the scale. The validated Norwegian translations of the MARS-5 and the BMQ were used in this study [19, 20].

Change in serum phosphate concentrations after the intervention was analysed with a linear mixed model, with a per patient random intercept to account for the repeated measurements before and after the intervention. A logistic mixed model was used to analyse the change in probability of serum phosphate concentrations being below 1.80 mmol/L. Linear mixed effects models were also used to analyse the effect of the intervention on the secondary outcomes. Correlations were also estimated with mixed effect models, where the serum phosphate concentrations were used as response and the secondary outcomes used as predictor variables. For these analyses the variables were standardized so that the coefficients could be interpreted as correlations. Associations between type of phosphate binder and patient knowledge and concerns before and after the intervention were tested using t-tests. Newcombs test of difference in proportions was used to test the associations between being on target (average across the five phosphate measurements being less than 1.8) and type of phosphate binder and type of dialysis. All analyses were done in R, using the lme4 package for the mixed effect models.

## Results

**Fig. 2:**
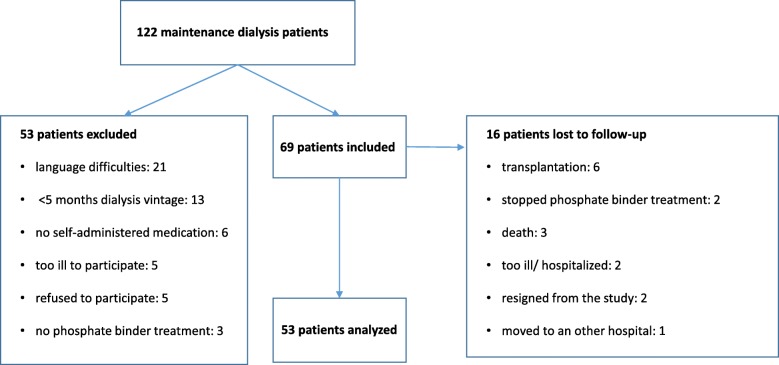
Inclusion of patients

At study start, 122 chronic patients attended the hospital dialysis centre. Of these, 74 met the eligibility criteria of which five patients refused to participate (Fig. 2). The main reason for not including patients was language difficulties, while kidney transplantation was the main reason for exclusion after enrolment. Demographic data for the 53 patients who completed the study are outlined in Table 2.

**Table 2:** Demographics of the study population (*n* = 53)

Characteristics	
Age in years, median, mean (range)	72, 68 (21–93)
Female, n (%)	16 (30)
Hemodialysis (HD), n (%)	37 (70)
Hemodiafiltration (HDF), n (%)	16 (30)
Dialysis months, median, mean (range)	28, 34 (4–140)
Number of medicines, median, mean (range)	13, 13 (7–20)
Patients using multidose drug dispensing aid^a^, n	11
Patients using N05^b^=psycholeptics and/or N06^b^=psychoanaleptics, n	25

^a^Multidose drug dispensing aid: meaning a sachet system where tablets and capsules for a particular date and dose time are packed in an individual sachet, labelled with date and time, the medicine details and the patient’s name. Sachets are prepared using automated packing technology [38]

^b^WHO Anatomical Therapeutic Chemical (ATC) classification**.** Psycholeptics = antipsychotics, anxiolytics, hypnotics and sedatives. Psychoanaleptics = antidepressants, psychostimulants and anti-dementia drugs

Sevelamer carbonate was the most commonly prescribed phosphate binder in the study group: as the only phosphate binder in 43% (*n* = 23) of the patients, and in combination with another phosphate binder in 30% (*n* = 16) of the patients. Lanthanum carbonate or calcium carbonate was prescribed as the only phosphate binder in 21% (*n* = 11) and 6% (*n* = 3) of the patients, respectively. No associations were found neither before nor after intervention between the type of phosphate binder in use and the mean number of correct answers for ‘Patient Knowledge’, or the mean scores for ‘Specific-Concerns’. Furthermore, the type of phosphate binder in use was not associated with having mean serum phosphate at target level.

The serum phosphate values in the study group are outlined in Table 3. Based on the mean values for each patient before and after the intervention, it was estimated that the proportion of patients with serum phosphate concentration below the target threshold value 1.80 mmol/L (5.58 mg/dL) increased from 45 to 49% in the total study group (odds ratio 1.27, 95% CI: 0.81, 1.98; *p* = 0.294, analysed by logistic mixed model). The mean levels were close to the target threshold 1.80 mmol/L (5.58 mg/dL) both prior to and after the intervention. No statistical change in serum phosphate was seen in the group after the intervention (mean change: 0.01 mmol/L (0.031 mg/dL), 95% CI:-0.04, 0.06; *p* = 0.593). A subgroup analysis of the patients (*N* = 29) with mean serum phosphate level > 1.8 mmol/L (5.58 mg/dL) at baseline showed an increase in the proportion of patients with serum phosphate < 1.80 mmol/L (5.58 mg/dL) after intervention (proportion increased from 0 to 22%, *N* = 6). However, no significant change was seen in mean serum phosphate after intervention (mean change: 0.05 mmol/L, 95% CI:-0.13, 0.03; *p* = 0.218).

**Table 3:** Serum phosphate values before and after intervention

Time^a^ relative to intervention (month)	Mean serum phosphate mmol/L (mg/dL) (mean)	Serum phosphate mmol/L (mg/dL) (range)	Serum phosphate mmol/L (mg/dL) (median)	Serum phosphate below 1.80 mmol/L (5.58 mg/dL) (proportion of patients)
−5	1.80 (5.58)	0.90–3.01 (2.79–9.33)	1.75 (5.43)	0.54
−4	1.85 (5.74)	1.03–2.99 (3.19–9.27)	1.83 (5.67)	0.45
−3	1.84 (5.70)	1.20–3.16 (3.72–9.79)	1.80 (5.58)	0.49
−2	1.83 (5.67)	1.06–2.79 (3.28–8.64)	1.82 (5.64)	0.47
−1	1.81 (5.61)	1.03–3.18 (3.19–9.85)	1.79 (5.55)	0.51
1	1.84 (5.70)	1.18–2.91 (3.65–9.02)	1.77 (5.49)	0.53
2	1.87 (5.80)	1.17–2.94 (3.62–9.11)	1.82 (5.64)	0.49
3	1.85 (5.74)	0.94–3.90 (2.82–2.09)	1.76 (5.46)	0.53
4	1.82 (5.64)	0.85–3.49 (2.63–0.81)	1.75 (5.43)	0.53
5	1.82 (5.64)	0.91–2.79 (2.82–8.64)	1.69 (5.24)	0.55

^a^Patients typically have one serum phosphate drawn each month. -5 = five months prior to intervention, 5 = five months after intervention

No associations were found neither before nor after intervention between the type of dialysis in use, hemodialysis (HD) versus hemodiafiltration (HDF), and the proportion of patients with mean serumphosphate at target level. The proportion of patients with mean serum phosphate at target level before intervention was 46% for those receiving HD (*N* = 37) and 44% for those receiving HDF (*N* = 16). After intervention, the proportion was 49% and 50% respectively.

The mean total scores for MARS-5 in the study group was high both before (22.1) and after (22.5) the intervention. No statistical change was seen in self-reported adherence after intervention measured by MARS-5 (mean change in total score: 0.415, CI: -0.304, 1.134; *p* = 0.258). Some improvement in self-reported adherence was seen after intervention measured by decrease in the proportion of patients who reported non-adherent behaviour for the statement “I take less than instructed”: 21% prior to and 11% after intervention. A patient was registered as non-adherent when “sometimes”, “often” or “always” was answered to the statement.

The total score for each BMQ subscale before and after intervention are outlined in Table 4. On average, patients’ beliefs in the necessity of phosphate binders were higher than their concerns both before and after the intervention. The study demonstrated a statistically significant increase in mean ‘Specific-Necessity’ scores and decrease in mean ‘Specific- Concern’ scores after intervention, while no change was seen in the mean scores for ‘General-Overuse’ and ‘General-Harm’. Also, there was a positive change in the ‘Necessity-Concern’ differential after intervention (mean change in scores: 2.5, CI: 1.201, 3.855; *p* = 0.001). The patients believed more in the importance of taking the phosphate binder and were less concerned about the side effects after the intervention.

**Table 4:** The Beliefs about Medicines Questionnaire (BMQ), scores before and after intervention

BMQ Subscale	Before intervention Mean score	After intervention Mean score	Change in score (95% CI)	*p* value
Specific-Necessity (5 items, score range 5–25)	18.2	19.2	1.0	0.005
-My health, at present, depends on my phosphate binder(s)	4.0	4.1	0.1	0.450
-My life would be impossible without my phosphate binder(s)	3.3	3.5	0.2	0.040
-Without my phosphate binder(s) I would be very ill	3.4	3.7	0.3	0.050
-My health in the future will depend on my phosphate binder(s)	3.6	3.8	0.2	0.150
-My phosphate binder(s) protect me from becoming worse	3.8	4.1	0.3	0.010
Specific-Concerns (6 items, score range 6–30)	15.3	13.8	−1.5	0.002
-Having to take my phosphate binder(s) worries me	2.3	2.1	−0.2	0.070
-I sometimes worry about the long-term effects of my phosphate binder(s)	2.9	2.5	−0.4	0.040
-My phosphate binder(s) are a mystery to me	2.9	2.5	−0.4	0.030
-My phoshphate binder(s) disrupt my life	2.5	2.2	−0.3	0.040
-I sometimes worry about becoming too dependent on my phosphate binder(s)	2.4	2.2	−0.2	0.050
-My phosphate binder(s) give(s) med unpleasant side effects	2.3	2.3	0.0	1.000
General-Overuse (4 items, score range 4–20)	11.4	11.8	0.4	0.177
- If doctors had more time with patients they would prescribe fewer medicines	3.1	3.2	0.1	0.030
-Doctors use too many medicines	3.0	3.0	0.0	1.000
-Doctors place too much trust on medicines	2.9	3.1	0.2	0.090
-Natural remedies are safer than medicines	2.4	2.4	0.0	1.000
General-Harm (4 items, score range 4–20	10.1	9.9	−0.2	0.624
-Most medicines are addictive	2.7	2.9	0.2	0.110
-Medicines do more harm than good	2.3	2.2	−0.1	0.450
-People who take medicines should stop their treatment for a while every now and again	2.5	2.2	−0.3	0.030
-All medicines are poisons	2.6	2.6	0.0	1.000

The mean number of scores for correct answers in the ‘Patient Knowledge’ questionnaire, in the total study group, was relatively low prior to the intervention: 5.0 of a possible maximum total score of 16.0. A positive change after the intervention was seen by a statistically significant increase in scores for correct answers (mean change in scores: 2.9, CI: 2.165, 3.570; *p* = 0.001). The mean number of wrong answers was 1.0 both before and after intervention, while the mean number of the answer “I do not know” decreased from 3.0 to 1.5 (mean change in number: -1.5, CI: -2.005, − 1.051; *p* = 0.001). Most frequently prior to intervention, the questions “When should you take the phosphate binder?” and “What should you do if you have forgotten to take the phosphate binder?” were answered correctly; 91% (48) and 79% (42) correct answers, respectively. Few patients knew the answer to the question “The phosphate binder may affect the uptake of the following medicines”; before intervention only six patients had one correct choice and no patients had more than one of a possible total of three correct choices to this question. The highest increase in correct choices after intervention was seen for the questions “For which condition are you using phosphate binder?”, “What can the use of phosphate binder prevent?” and “Which of these foods/drinks are especially rich in phosphate?”. The mean number of correct choices for each question adjusted for the number of possible correct choices, increased from 0.51, 0.19 and 0.52 to 0.77, 0.42 and 0.76 respectively.

## Discussion

This study shows that education and counselling about the use of phosphate binders have a positive effect on the dialysis patients’ knowledge about the medicines, increase their sense of necessity of the treatment and reduce their concerns. This positive effect was reflected in an increased probability of serum phosphate being below the target threshold value 1.80 mmol/L (5.58 mg/dL), although not statistically significant. The intervention did not affect the mean serum phosphate in the total study group nor the self-reported adherence.

The limited effect on serum phosphate and adherence could be due to the intervention being insufficient, including one information session only. Van Camp et al [11] investigated the effect of a 13-week nurse-led education and counselling on the adherence to phosphate binders. Adherence was evaluated before and after the intervention, which included both education about phosphate binders and thereafter bi-weekly individual counselling. They found that the intervention led to increase in adherence from 83 to 94% in the intervention group, while in the control group the adherence decreased from 86 to 76%. Moreover mean serum phosphate levels decreased from 4.9 to 4.3 mg/dL (1.58 to 1.38 mmol/L). Another explanation to this discrepancy in results could be the power of our study being insufficient. It was, however, not possible to extend the study population beyond the limit of the patient baseline of 120, as the patients were included during a limited period of time, to avoid spill-over effect. Also, it was not possible to perform a randomised controlled study because of the proximity of patient chairs during the intervention.

Individual variability in pharmacological response to the phosphate binder, diet and absorption of phosphate, in addition to the individual variety in residual kidney function and dialytic removal of phosphate will influence on the level of serum phosphate [21–25]. This study does not include measurable variables such as vitamin D use, cinacalcet use, phosphate binder dosing, diet and changes in such variables that could have affected the serum phosphate levels during the study period.

Apparently, the self-reported adherence in this study was high (> 22) both before and after intervention, which gives little room for measuring improvement by the MARS-5 scale. The realtively high level of self-reported adherence seems to be in conflict with results from other studies stating that non-adherence is prevalent in dialysis patients, although a wide variation in rates of non-adherence is reported. The variation can be explained by differences in definitions and methods [4, 6]. There is an inconsistency in the published literature regarding the use of cut-off limits defining patients as adherent or non-adherent. In this study, no specific cut-off limit was defined in accordance with the method described by Horne and Weinman [8]. Others have defined adherence as a MARS-5 score of > 20 or > 23 [26, 27].

Van Camp et al [11] measured adherence for 17 consecutive weeks using the electronic Medication Event Monitoring System (MEMS®). In addition, pill-count and self-report were used to corroborate the MEMS® data. Evaluation of adherence over time was investigated and compared with historical data from an observational cohort study. In the intervention group adherence increased during the study period, while in the control group adherence declined. In our study, self-reported adherence was not validated by counting of tablet or other objective methods, which adds an uncertainty when interpreting the scores from MARS-5. Patients might feel pressured to provide a desirable response rather than an accurate assessment of their adherence [10]. Also, the study pharmacist uncovered examples of misunderstandings potentially influencing the results, like a patient who thought the phosphate binder was a painkiller, and did not take it because he did not have any pain. This patient was under the impression that he did not have phosphate binders on his medication list. These findings also highlights the issue regarding the sensitivity of MARS-5 as a tool for measuring adherence in this patient group.

The aforementioned patient was using multi-dose drug dispensing aid (MDD). Multi-dose drug dispensing is offered to patients, mostly elderly, with regular medication use combined with difficulties in handling and administering their drugs. The patients get their drugs machine dispensed into one unit bag for each dose occasion. The dose unit bags are labeled with patient data, drug contents, date and time for intake [28]*.* MDD users report more remoteness of their drugs and less knowledge about the indications for their drugs compared to non-MDD users [29]. The patient’s lack of knowledge about the indications and his inability to identify the tablets in the MDD, lead the patient to poor adherence and to unintentionally not take the phosphate binders he needed. Individual counselling of the patient revealed this lack of knowledge and gave the patient the information he needed to make a knowledge-based choice and take the phosphate binders as intended.

Patients may be intentionally or non-intentionally non-adherent to treatment [23, 30–32]. During counselling, study patients reported challenges such as forgetfulness, denial of necessity, social embarrassment by taking tablets in company with others, impracticality with bringing tablets/ not having a proper container for bringing tablets, discomfort (taste, not allowed to swallow with fluid etc.), tablet burden and side effects (nausea, diarrhoea, constipation, stomach ache). One example of intentional non-adherence, expressed by some patients, was the decision that quality of life was more important than living longer. Cognitive function has been demonstrated in the literature to be lower while the patient is receiving dialysis than in between dialysis sessions [33]. For practical reasons like lack of facilities and logistic challenges, we could not administer the questionnaires prior to dialysis. This might have affected the patients understanding of the questionnaires and their ability to fill out the survey accurately. The majority of patients in our study were male (70%) more than 60 years old (83%), who had been treated with dialysis for a long time (mean; 34 months). Cognitive impairment in dialysis patients is well documented and could influence the effect of the intervention and overall adherence [34–37]. Considering the relatively high median age (72) of the study population and the possible cognitive impairment, scores for cognitive function could have been part of the inclusion criteria. Wileman used mini mental status evaluation (MMSE) with a certain score as inclusion criteria [23]. However, excluding patient with these challenges would have given overoptimistic results regarding effect, as cognitive impairment is common in the study population. During counselling, some of the patients told that their family members helped them with their medicines and reminded them to take the phosphate binder. This implies that relatives should be given the same information as the patients regarding medication treatment.

Educational interventions may have the greatest effect immediately following intervention and then this may wane over time. We assessed the questionnaires pre-intervention and then up to 4 weeks post-intervention. An assessment of the questionnaires immediately following intervention might have shown higher scores. In the perspective of patient safety and benefit, assessment of the questionnaires after a period of time provide a more realistic measurement of effect.

Our study increased the patients’ level of knowledge on how to control their phosphate levels, especially when it comes to why they should take their phosphate binders. This knowledge might be important for patients’ motivation for being adherent.

## Conclusions

Short term one-to-one individualized pharmacist-led education and counselling about phosphate binders increased the probability of serum phosphate concentrations being below the target threshold level 1.80 mmol/L (5.58 mg/dL), although not statistically significant. However it did not decrease the mean serum phosphate level or increase the patients’ self-reported adherence. The patients increased their knowledge about the phosphate binder and their understanding of adherence, and were less concerned about the side effects of the medication.

## Abbreviations

BMQ: Beliefs about Medicines Questionnaire
CKD: Chronic Kidney Disease
MARS-5: Medication Adherence Report Scale
MDD: Multidose Drug Dispensing
MEMS®: Medication Event Monitoring System
MMSE: Mini Mental Status Evaluation
